# Joint-specific assessment of swelling and power Doppler in obese rheumatoid arthritis patients

**DOI:** 10.1186/s12891-017-1406-7

**Published:** 2017-03-04

**Authors:** Erin M. Bauer, Ami Ben-Artzi, Erin L. Duffy, David A. Elashoff, Sitaram S. Vangala, John Fitzgerald, Veena K. Ranganath

**Affiliations:** 1 Department of Medicine, David Geffen School of Medicine, University of California, Los Angeles, UCLA, Los Angeles, CA USA; 2 U.S. Department of Veterans Affairs, Los Angeles, CA USA; 3 Cedars-Sinai Medical Center, Los Angeles, CA USA

**Keywords:** Ultrasound, Obesity, Outcome measures, Rheumatoid arthritis

## Abstract

**Background:**

Clinical swollen joint examination of the obese rheumatoid arthritis (RA) patient can be difficult. Musculoskeletal Ultrasound (MSUS) has higher sensitivity than physical examination for swollen joints (SJ). The purpose of this study was to determine the joint-specific association between power Doppler (PDUS) and clinical SJ in RA across body mass index (BMI) categories.

**Methods:**

Cross-sectional clinical and laboratory data were collected on 43 RA patients. PDUS was performed on 9 joints (wrist, metacarpalphalangeal 2–5, proximal interphalgeal 2/3 and metatarsalphalangeal 2/5). DAS28 and clinical disease activity index (CDAI) were calculated. Patients were categorized by BMI: <25, 25–30, and >30. Demographic and clinical characteristics were compared across BMI groups with Kruskal-Wallis test and chi-square tests. Joint-level associations between PDUS and clinically SJ were evaluated with mixed effects logistic regression models.

**Results:**

While demographics and clinically-determined disease activity were similar among BMI groups, PDUS scores significantly differed (*p* = 0.02). Using PDUS activity as the reference standard for synovitis and clinically SJ as the test, the positive predictive value of SJ was significantly lower in higher BMI groups (0.71 in BMI < 25, 0.58 in BMI 25–30 and 0.44 in BMI < 30) (*p* = 0.02). The logistic model demonstrated that increased BMI category resulted in decreased likelihood of PDUS positivity (OR 0.52, *p* = 0.03).

**Conclusions:**

This study suggests that in an obese RA patient, a clinically assessed SJ is less likely to represent true synovitis (as measured by PDUS). Disease activity in obese RA patients may be overestimated by CDAI/DAS28 calculations and clinicians when considering change in therapy.

**Electronic supplementary material:**

The online version of this article (doi:10.1186/s12891-017-1406-7) contains supplementary material, which is available to authorized users.

## Background

Musculoskeletal ultrasound (MSUS) with power Doppler (PDUS) has become an accepted modality to identify features of inflammatory arthritis including synovitis and enthesitis and expert panels from the American College of Rheumatology (ACR) and the European League Against Rheumatism (EULAR) consider its use reasonable for monitoring disease activity in patients with rheumatoid arthritis (RA) [1, 2]. Synovitis is the manifestation of synovial proliferation and angiogenesis, which is an early pathologic change leading to bony destruction by locally invading the synovium-cartilage interface [3]. The Outcome Measures in Rheumatoid Arthritis Clinical Trials (OMERACT) initiative defines synovitis by PDUS as abnormal hypoechoic intraarticular tissue that is non-displaceable, poorly compressible, and exhibiting Doppler signal [4]. Unlike healthy synovium, inflamed synovium is hyperemic and can be semi quantitatively graded, most commonly on a 4-point scale of 0 to 3 for PDUS activity [5]. Multiple studies measuring PDUS have documented improvement of PDUS scores in response to RA treatments [6, 7]. PDUS is reported to have higher sensitivity and be more reliable for synovitis evaluation than an examination of swollen joints [8] and is predictive of relapse and radiographic disease progression [9].

While the uses and benefits of MSUS in RA are becoming widely recognized, the burden of time and cost can be prohibitive in obtaining MSUS on every RA patient within the rheumatologists’ clinical practice. It may be beneficial to better define the type of RA patient and clinical scenario in which MSUS will have differential impact on therapeutic management especially given the push towards early and aggressive control of disease activity. The majority of RA patients in the United States are considered overweight or obese [10, 11], and these patients have a poorer quality of life [12] and are less likely to attain remission by Disease Activity Score (DAS28) <2.6 despite treatment as compared to non-obese RA patients [13, 14]. This lack of measured response in obese RA patient could be due to a differential response to therapy, a measurement error in assessing disease activity, or a combination of both. PDUS in the obese RA patient has not been fully described in the literature to date.

Our objective in this study was to evaluate the joint specific association between synovitis measured by PDUS and the clinically swollen joint in overweight and obese RA patients.

## Methods

### Patients

Patients meeting the ACR 1987 and ACR/EULAR RA diagnostic criteria [15, 16] and whose treating physicians were considering switching and/or escalating immunosuppressive therapy were recruited from University of California at Los Angeles (UCLA) rheumatology clinics. Patients were required to have active RA, with a minimal swollen joint count (SJC) ≥ 2 and tender joint count (TJC) ≥ 2 of the 28 joint count examined. Eligible RA patients met the following criteria: age ≥ 18, stable disease modifying anti-rheumatic drugs (DMARDs), and on prednisone ≤ 10 mg for at least one month. Patients were excluded if they were pregnant or breast feeding, or if they had arthroplasty of the joints examined by ultrasound. Based on these inclusion/exclusion criteria, 43 patients were evaluated. These patients were then divided into three groups based on their body mass index (BMI): <25, 25 to 30, and >30 for analysis. The study was approved by the UCLA institutional review board (IRB#11-001225-CR-00005).

### Clinical study measures

Patients completed demographic questionnaires, and the components of the DAS28 and Clinical Disease Activity Index (CDAI) were obtained the same day as the ultrasound. The erythrocyte sedimentation rate (ESR) was measured to calculate the DAS28/ESR. Rheumatoid factor (RF) and anti-cyclic citrullinated protein antibody (ACPA) were obtained for each patient. The total tender and swollen joint count was assessed by a single study investigator (VKR) prior to ultrasound.

### Musculoskeletal ultrasound synovitis measures

MSUS assessment of PDUS and grey scale (GSUS) hypertrophy and effusion was performed at the one-time visit. The sonographer (ABA) was an experienced rheumatologist trained in MSUS and certified in Rheumatology MSUS (rhMSUS). Images were obtained using a GE Logic E9 machine with ML6-15 linear probe and the following presets: red-yellow color map, Doppler frequency 10.0 MHz, PRF 0.8KHz, and gain adjusted just below noise. Based on a prior publication by Backhaus et al [6], with the addition of 2 joints (metacarpalphalangeal 4 and 5), a total of 9 joints were scanned by MSUS on the most active side: wrist (dorsal longitudinal midline view), metacarpalphalangeal (MCP) joints 2, 3, 4, and 5 (dorsal/palmar long views), proximal interphalgeal (PIP) joints 2 and 3 (dorsal/palmar long views) and metatarsalphalangeal joints 2 and 5 (dorsal long view). PDUS and GSUS were scored semi-quantitatively on a scale of 0–3 per prior published consensus definitions [6]. Specifically, PDUS was scored by the amount of power Doppler signal in the intraarticular area: grade 0 = no color signal, grade 1 = up to 3 color signals or 2 single and 1 confluent signal, grade 2 = greater than grade 1 to < 50% color signal, and grade 3 = >50% color signal [6]. The max score of the views obtained for each joint was computed and then was theses maximums were summed across all 9 joints to obtain total PDUS (range 0–27) and GSUS (range 0–27) scores. An individual joint was considered to be positive for synovitis if its max PDUS score was ≥1. The clinical assessor was blinded to the ultrasound data, and the ultrasonographer was blinded to the clinical assessments. Additionally, when scoring the archived images, the ultrasonographer was blinded to all clinical data. Once all scans were completed the ultrasonographer re-read 10% of randomly selected ultrasound visits for kappa intra-rater reliability calculations for GSUS and PDUS [17].

### Statistical analysis

Patients’ mean (standard deviation [SD]) age and disease duration were calculated for each BMI group and compared across groups with t-tests. The proportion of female and sero-positive (RF+ or ACPA+) patients was calculated for each BMI group and compared across groups with Fisher Exact Tests. The GSUS, PDUS, SJC, TJC, DAS28/ESR-4, CDAI, and ESR for each BMI group were compared across BMI groups with Kruskal-Wallis tests. Kruskal-Wallis Tests were used for these US and clinical measures because they exhibited skewed distributions in visual assessments.

Agreement between clinical swollen joint assessments and PDUS assessments were evaluated for the full cohort and each BMI group in several ways. First, the overall percent of joints that were considered swollen and PDUS positive were calculated for the full cohort and each BMI group. Next, the percent of joint-specific pairwise agreement between swollen joint assessment and PDUS assessment was calculated. To assess the predictive relationship of clinical swollen joints versus PDUS positivity (reference standard), the sensitivity, specificity, positive predictive value (PPV), and negative predictive value (NPV) of swollen joint assessment were calculated.

We constructed a mixed effects logistic regression model to evaluate the correlates of PDUS positivity, as well as for GSUS. This model included terms for SJC, BMI group (represented ordinally), age, sex, joint, as well as a random effect for subject to account for the clustering of joints within subjects. Within the logistic regression, BMI was represented as an ordinal variable by converting the 3 categories of BMI as follows: BMI <25 = 1, BMI 25–30 = 2, and BMI > 30 = 3. To evaluate the predictive ability of this model for PDUS positivity, we constructed a ROC curve using the predicted probabilities from the model and computed the area under the ROC curve (AUC). Lastly, we included a term in the model for specific joint to evaluate in which joints the differences between BMI groups were most pronounced.

## Results

Demographic characteristics were similar among BMI groups. The overall cohort was 86% female and 66% ACPA or RF seropositive with a mean age of 52.1 (SD 12.9) and disease duration of 7.3 (SD 7.8) years (Table 1). Within the cohort 93% of patients were on a DMARD, 19% were on Biologics, and 16% of patients were on prednisone. There were no significant differences in these characteristics across the BMI categories.

**Table 1:** Baseline demographics, mean (SD) or number (%)

	Overall	BMI <25		BMI 25–30		BMI >30		*P*-value
	*N* = 43	*N* = 17		*N* = 12		*N* = 14		
BMI^1,^*	28.9 (9.0)	21.3	(2.3)	27.2	(1.4)	39.5	(7.3)	
Age (years)*	52.1 (12.9)	49.9	(16.9)	56.6	(7.5)	51.1	(10.5)	0.37
Disease duration (years)*	7.3 (7.8)	7.4	(9.6)	4.0	(3.8)	10.1	(7.2)	0.14
Female**	37 (86%)	17	(100%)	8	(67%)	12	(86%)	0.02
RF^2^ or CCP^3^ Positive**	27 (66%)	11	(69%)	9	(75%)	7	(54%)	0.55
DMARD (Y)	40 (93%)	1	(6%)	0	(0%)	2	(14%)	0.48
Biologics (Y)	8 (19%)	2	(12%)	2	(17%)	4	(28%)	0.55
Prednisone (Y)	7 (16%)	3	(18%)	2	(17%)	2	(14%)	0.99

^1^Body Mass Index, ^2^Rheumatoid Factor, ^3^Cyclic citrullinated peptide, *Mean (SD) and one-way ANOVA, **N (%) and Fisher Exact Test

The overall median and interquartile ranges (IQR): 4 (2) for SJC, 4 (4) for TJC, 6.3 (1.5) for DAS28/ESR-4, and 36.5 (29.0) CDAI score (Table 2). These disease activity as measures were not significantly different across the BMI groups. While GSUS was not significantly different across BMI groups (*p* = 0.42), the PDUS scores significantly differed across BMI groups with median (IQR) scores of 3 (3) for BMI group <25, 1 (3.5) for BMI group 25–30 and 0 (2) BMI group >30 (*p* = 0.02).

**Table 2:** Musculoskeletal ultrasound synovitis measures and disease activity measures, median IQR (25%, 75%)

	Overall	BMI <25		BMI 25–30		BMI >30		*P*-value
	*N* = 43	*N* = 17		*N* = 12		*N* = 14		
SJC	4 (3, 5)	5	(3, 5)	4	(2, 4.5)	4	(3, 5)	0.40
TJC	4 (2, 6)	4	(3, 6)	3	(2, 5)	4	(2, 6)	0.57
ESR	36.5 (25, 54)	37	(30, 54)	35	(24, 52)	40	(25, 54)	0.77
DAS28/ESR-4	6.3 (5.4, 6.9)	6.6	(5.4, 7.0)	6.2	(5.5, 6.5)	6.1	(5.2, 6.9)	0.44
CDAI	36 (26, 43)	36	(30, 44)	35	(21.5, 42)	35	(26, 42)	0.67
GSUS	5 (3, 6)	5	(3, 6)	4	(2.5, 6)	5.5	(4, 6)	0.42
PDUS	1 (0, 3)	3	(1, 4)	1	(0, 3.5)	0	(0, 2)	0.02

GSUS-Gray Scale Ultrasound, PDUS-Power doppler Ultrasound, SJC-Swollen Joint Count, TJC-Tender Joint Count, CDAI-Clinical Disease Activity Index, ESR-Erythrocyte Sedimentation Rate. Kruskal-Wallis tests used to compare groups

**Fig. 1:**
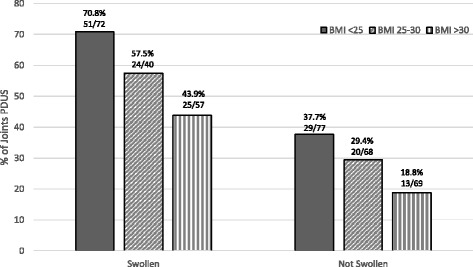
The proportion of PDUS positivity stratified by BMI category and swollen joint status. PDUS: power Doppler ultrasound, BMI: body mass index

The prevalence of PDUS positive joints decreased across BMI categories (54% for BMI < 25, 40% for BMI 25–30, and 30% for BMI > 30), while prevalence of swollen joints in the high BMI group was only 3 percentage points lower than in the low BMI group (48% for BMI < 25 vs 45% for BMI > 30) (Table 3). Using PDUS activity as the reference standard for synovitis and SJC as the test (joint-specific analyses), the positive predictive value (PPV) of SJC was significantly lower in higher BMI groups (0.71 in BMI < 25, 0.58 in BMI 25–30 and 0.44 in BMI > 30, *p* = 0.02). The negative predicative value (NPV) trended higher in >30 BMI group (0.62 for BMI group <25, 0.71 for BMI group 25–30, 0.81 in BMI group >30) although was not statistically significant. In addition, clinically swollen joints had higher rates of PDUS compared to non-swollen joints, and there was a decreasing trend of PDUS positivity with increasing BMI for both swollen and non-swollen joints (Fig. 1). For example, a clinically swollen joint in a RA patient with normal BMI had 71% chance of PDUS positivity, whereas a swollen joint in an obese patient had a 44% chance. On the other hand, a non-swollen joint in a normal BMI patient still had a PDUS positivity rate of 38%, while lower in the obese patient’s non-swollen joint (19%).

**Table 3:** PDUS and Swollen Joint Agreement by BMI Group

	Overall	BMI < 25	BMI 25–30	BMI > 30	*p*
% Joints PDUS Positive	42%	54%	40%	30%	0.018
% Joints Swollen	44%	48%	37%	45%	0.611
% Agreement	66%	66%	66%	64%	0.713
Sensitivity	0.61	0.64	0.53	0.66	0.822
Specificity	0.68	0.70	0.74	0.64	0.691
PPV	0.59	0.71	0.58	0.44	0.023
NPV	0.71	0.62	0.71	0.81	0.084

*PPV* Positive Predictive Value, *NPV* Negative Predictive Value

**Fig. 2:**
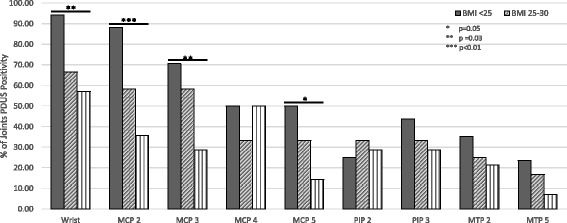
Percentage of PDUS positivity stratified by joint and BMI category

The logistic regression model demonstrates that each BMI category increase is independently associated with lower odds of PDUS positivity with an odds ratio of 0.52 (95% CI: 0.08, 0.88, *p* = 0.03), adjusting for covariates (Table 4). Additionally, the logistic model shows that the presence of clinical joint swelling is positively associated with the likelihood of PDUS positivity (OR = 2.5, 95% CI: 1.4 to 4.5, *p* = 0.01). The AUC of the logistic model was 0.76 suggesting the combination of BMI, clinical swollen joint assessment, sex and age provide strongly predict the presence of PDUS positivity. Additionally, we constructed a similar logistic model for outcome of GSUS positivity and found similar results for the association of clinical swelling and GSUS (OR = 2.52) to what was observed in the PDUS model (Table in Additional file 1). In particular, the wrist, MCP2, and MCP3 had significantly less PDUS positivity in the BMI > 30 group as compared to the BMI < 25 group (Fig. 2). The ultrasonographer’s intra-rater reliability for GSUS and PDUS was a kappa of 0.62 and 0.82, respectively.

**Table 4:** Multivariate Logistic model for PDUS (accounts for correlation among different joints in the same patient)

	Odds Ratio	95% CI Lower	95% CI Upper	*P*-value
Age	1.01	0.98	1.05	0.48
Sex (Female)	2.42	0.54	10.87	0.25
BMI (Ordinal)	0.52	0.30	0.93	0.03
Swollen (Yes)	2.50	1.40	4.48	0.01

*PDUS* power Doppler ultrasound

## Discussion

Despite continuing advances in therapeutic options for RA, almost 30% of all RA patients will fail to meet the goals of quick and sustained control of disease activity with a first exposure to a biologic agent and will need to switch therapy [18]. Given the emphasis on early intervention to prevent the destructive changes in RA and the push to “treat to target” (T2T) by national and international guidelines [19–21], there is incentive to know when a patient is not responding to a given treatment. The current gold standard for monitoring disease activity and thus the “target” of T2T is usually DAS28 remission (<2.6) or low activity (<3.2). While the majority of T2T studies and guidelines employ DAS28, CDAI, or Simple Disease Activity Index (SDAI), there are criticisms that these targets require prior blood work (DAS28, SDAI), do not account for the joints in the feet, and that the tender joint counts can be misleading for disease activity measures due to attribution error in patients with co-morbidities such as fibromyalgia and osteoarthritis. Our study addresses the latter point. Other studies have suggested that RA patients with fibromyalgia were found to have elevated disease activity scores, however noted to have low or absent measures of clinical synovitis or MSUS synovitis [22, 23]. Our study is the first to our knowledge, which investigates the discordance of elevated disease activity scores in obese patients and absent/low measureable synovitis by PDUS.

The obese and overweight patients with RA pose a considerable management challenge. Studies report that obese and overweight patients with RA are less likely to attain disease remission [24–26] and are more likely to have limited therapeutic response [13] as compared to non-obese RA patients, even with weight-adjusted treatments [14]. Sandberg et al found a significant dose response relationship between a BMI and change in disease activity in a study of 495 Swedish patients. Those with a BMI ≥ 25 had a 51% lower odds of reaching low disease activity as measured by DAS28 after 6 months of follow up (OR = 0.49 95%CI 0.31 to 0.78) [18]. Obese RA patients also have higher rates of functional disability, cardiovascular risks, and decreased quality of life as compared to non-obese RA patients [27, 28]. Interestingly, although overweight patients have a decreased chance of achieving good disease control as measured by DAS28, multiple studies have suggested that patients with higher BMIs may have less radiographic joint damage [29], in particular in ACPA positive obese patients [30]. Thus, the natural question that arises is whether there could be a potential measurement error of disease activity in obese RA.

Our study implies that clinically assessed swollen joints are less likely to represent true synovitis in obese RA patients. Therefore, in obese patients, RA disease activity can be overestimated by CDAI and DAS28 calculations which may help to explain the reports that obese and overweight patients with RA are less likely to attain remission and are more likely to have limited therapeutic response as compared to non-obese RA patients.

Multiple studies have reported that increased BMI is associated with less radiographic joint damage (total erosions, joint space narrowing) in RA [29–31]. Theories to explain this have invoked the protective effects of adipocytes on synovial tissue, decreased hydroxypyridinium collagen crosslinking, the anti-inflammatory properties of adiponectin and estrogen [32] and beneficial bone remodeling secondary to greater weight [29, 33]. Our study suggests that obese RA patients may simply have less synovitis as compared to non-obese patients with the same calculated disease activity scores. This is an alternate possible explanation for prior observations that obese patients who have a less severe disease course radiographically as compared to non-obese matched controls. The assumption that joint destruction in RA is related to synovial hyperemia is supported by several studies including a 2008 study by Naredo et al which included MSUS with PDUS of 367 patients with RA and demonstrated that PDUS signal was predicative of radiographic erosions and progression on plain films (R = 0.64) [34]. In addition, studies using bone marrow edema as seen on magnetic resonance imaging (MRI) to assess for disease activity have shown similar results. In a 2014 Baker et al study, a secondary analysis of the GO-BEFORE and GO-FORWARD randomized clinical trials, evaluated the efficacy of golimumab in combination with methotrexate compared with methotrexate and golimumab monotherapy and demonstrated that there was a significantly negative correlation between bone edema score and BMI at baseline [33].

Both ACR and EULAR support the use of MSUS to monitor RA disease activity and this study suggests that it may be a particularly important tool in the evaluation and management of the obese patient. In the United States 60–70% of RA patients are overweight or obese [10, 11]. Therefore, assessment of PDUS could be considered in the majority of our RA patients in order to gain a better understanding of their true disease activity. Prior studies have suggested that MSUS with PDUS is more sensitive than physical examination in detection of synovitis [8] which is consistent with our study as obese RA subjects with clinically swollen joints have a lower PPV and a trend for higher NPV for true synovitis, while the opposite is found for normal BMI patients.

Frequent MSUS assessments require an experienced and consistent ultrasonographer and is a time consuming process if a large number of joints are examined. Recent studies have suggested that the addition of MSUS to management of an *early* RA patient population provides no additional effect compared with a conventional tight control strategy [35, 36]. However, if the correct established RA patient population can be targeted, the costs of MSUS may off set the burdens associated with switching/escalating pharmacologic therapies including support/authorization staff time, adverse events and side effects of immunosuppressive medications. Our study suggests that of all the joints included, the wrist, MCP2 and MCP 3 show the greatest discrepancy in swollen joint count by exam and PDUS activity between patients with BMI > 30 and patients with BMI < 25. Further study could lead to the ability to perform a more targeted exam.

This study is limited by a relatively small sample size, however, several of our analyses included a larger number of joint-specific pairwise agreement assessments between swollen joint and PDUS. While the baseline patient characteristics were similar across all BMI groups there were no men in the BMI < 25 group. This was a cross-sectional study and thus we are unable to examine the differential effect, if any, on treatment response in patients who are overweight or obese. Other patient co-morbidities such as osteoarthritis and fibromyalgia were not obtained. Aside from ESR and CRP we did not measure markers that others have correlated with obese patients such as adiponectin and leptin. We chose MSUS with PDUS as our gold standard for synovitis while some may consider MRI the gold standard. However, MRI is limited in the number of joints that can be assessed and requires contrast and, studies have demonstrated a high level of agreement between MSUS with PDUS and MRI for detection of synovitis [37, 38] with at least one study reporting that US was more sensitive than MRI in the detection of synovitis [39]. Additionally, as mentioned above, there is evidence that obese patients have significantly less synovitis as measured by RA MRI (RAMRIS) synovitis score as compared to patients with BMI <30 despite having similar disease activity scores measured by DAS28(CRP) which is consistent with our findings [33]. Lastly, we noted there was a trivial difference in the depth from skin to joint in the obese patients, but only at the wrist (0.1 mm), versus those patients who were overweight or normal. This difference was not significant enough to explain lower scores (by way of beam penetration) in the obese patients.

## Conclusion

This study suggests that in a cohort of RA patients whose rheumatologist was considering switching therapy, obese RA subjects had lower PDUS scores than non-obese patients while having similar clinical disease activity scores. In addition, BMI ≥ 30 was an independent predictor of lower PDUS while accounting for other factors. Obese RA patients’ clinically swollen joints had a lower PPV and a higher NPV for true synovitis. This implies that clinically assessed swollen joints are less likely to represent true synovitis in obese RA patients, suggesting that RA disease activity can be overestimated by CDAI and DAS28 calculations. With the push towards “treating to target” of remission or low disease activity by DAS28 or CDAI alone, the BMI of the patient should taken in to account. If there is a question of whether a joint is swollen, MSUS with PDUS could be considered prior to changing or escalating therapy.

## Additional file

Additional file 1: Obesity US Manuscript Supplemental Material. Multivariate Logistic model for GSUS (accounts for correlation among different joints in the same patient). (DOCX 12 kb)

## Abbreviations

ACPA: Anti-cyclic citrullinated protein antibody
ACR: American College of Rheumatology
BMI: Body mass index
CDAI: Clinical disease activity index
DAS: Disease Activity Score
DMARD: Disease modifying anti-rheumatic drug
ESR: Erythrocyte sedimentation rate
EULAR: European League Against Rheumatism
GSUS: Grey scale synovial hypertrophy
IQR: Interquartile range
MCP: Metacarpalphalangeal
MSUS: Musculoskeletal ultrasound
NPV: Negative predictive value
OMERACT: Outcome Measures in Rheumatoid Arthritis Clinical Trials
PDUS: Power Doppler ultrasound
PIP: Proximal interphalgeal
PPV: Positive predictive value
PPV: Positive predictive value
RA: Rheumatoid arthritis
RF: Rheumatoid factor
SD: Standard deviation
SDAI: Simple Disease Activity Index
SJ: Swollen joints
SJC: swollen joint count
T2T: Treat to target
TJC: Tender joint count
UCLA: University of California at Los Angeles.
