# Supplementary material for: Joint-specific assessment of swelling and power Doppler in obese rheumatoid arthritis patients
Source: BMC Musculoskelet Disord. 2017 Mar 4;18:99. doi: 10.1186/s12891-017-1406-7 (PMC5336673; doi:10.1186/s12891-017-1406-7)
Supplement: Additional file 1: — Obesity US Manuscript Supplemental Material. Multivariate Logistic model for GSUS (accounts for correlation among different joints in the same patient). (DOCX 12 kb) [file 12891_2017_1406_MOESM1_ESM.docx]

| **SUPPLEMENTAL MATERIAL**  Table 5: Multivariate Logistic model for GSUS (accounts for correlation among different joints in the same patient) | | | | |
| --- | --- | --- | --- | --- |
|  | Odds Ratio | 95% CI Lower | 95% CI Upper | P-value |
| Age | 0.997 | 0.976 | 1.020 | 0.817 |
| Sex (Female) | 1.315 | 0.633 | 2.732 | 0.463 |
| BMI (Ordinal) | 0.826 | 0.596 | 0.145 | 0.253 |
| Swollen (Yes) | **2.518** | **1.335** | **4.750** | **0.005** |
